# Extract from phyllosphere bacteria with antibiofilm and quorum quenching activity to control several fish pathogenic bacteria

**DOI:** 10.1186/s13104-021-05612-w

**Published:** 2021-05-25

**Authors:** Olivia Nathalia, Diana Elizabeth Waturangi

**Affiliations:** Faculty of Biotechnology, Atma Jaya Catholic University of Indonesia, Jalan Jenderal Sudirman 51, Jakarta, 12930 Indonesia

**Keywords:** Antibiofilm, Phyllosphere bacteria, Quorum sensing, Quorum quenching, Fish pathogen

## Abstract

**Objective:**

The objective of this research were to screen quorum quenching activity compound from phyllosphere bacteria as well as antibiofilm activity against several fish pathogen bacteria such as *Aeromonas hydrophila*, *Streptococcus agalactiae*, and *Vibrio harveyi*.

**Results:**

We found eight phyllosphere bacteria isolates with potential quorum quenching activity to inhibit *Chromobacterium violaceum* as indicator bacteria. Crude extracts (20 mg/mL) showed various antibiofilm activity against fish pathogenic bacteria used in this study. Isolate JB 17B showed the highest activity to inhibit biofilm formation of *A. hydrophila* and *V. harveyi*, meanwhile isolate JB 3B showed the highest activity to inhibit biofilm of *S. agalactiae*. From destruction assay, isolate JB 8F showed the highest activity to disrupt biofilm of *A. hydrophila* isolate JB 20B showed the highest activity to disrupt biofilm of *V. harveyi*, isolate JB 17B also showed the highest activity to disrupt biofilm of *S. agalactiae.*

## Introduction

The high production of aquaculture has contributed to the development of the economy of Indonesia when there was a significantly increased demand for marine products, such as fish. However there are problems in this field need to be handled including infectious diseases caused by several pathogenic bacteria. Antibiotic therapy is a major treatment for these diseases, but incidence of antibiotic resistance in pathogenic bacteria is increasing, on other hand the use of antibiotics can disrupt the presence of intestinal microbiota communities and trigger a population of bacteria becoming resistant and effects on public health [1, 2]. A lot of research reported several pathogenic bacteria capable form biofilm to protect their cell from environmental stresses and antibiotics exposure. Therefore exploration of antibiofilm agents is important. The most studied regulatory mechanism that has been found to control biofilm formation is quorum sensing regulation [3]. Quorum sensing is a process in which bacteria can secrete specific extracellular signalling molecules called autoinducer [4].

Medicinal plants have been studied possess therapeutic properties in infectious disease treatments caused by pathogenic bacteria. Microbiota on the leaves surface contribute important role in these properties. There are various kinds of microbial communities including bacteria, fungi, as well as algae on the surface of leaves, these microbes live in interaction with plants by using plant’s exudate as for their nutritional resources, while in return microbe also contribute to support the growth and health of plants. Bacteria are known as the most abundant inhabitants of the phyllosphere. Compound from phyllosphere bacteria have been used as biocontrol to prevent plant from infectious diseases causing by pathogenic bacteria, these compound degrade molecule signal (AHL) of pathogenic bacteria therefore inhibit their infection [5]. Most of the compound isolated from phyllosphere bacteria with anti quorum sensing activities are lactonase. Another study showed the first enzyme was identified from *Bacillus* sp. Strain 24B01, and the gene encoding the enzyme was named *aiiA* for AI inactivation and its protein showed capability in inactivation of AHL signals through hydrolysis of the lactone ring [6]. Similarly, *aiiD* gene in *P. aeruginosa* also showed activity decreased the concentration of AHL, this bacteria produce extracellular proteolytic enzymes such as lactonase and acylase and breakdown the AHL molecules [7]. In fact, phyllosphere bacteria can act as phytopathogen or inhibit phytopathogen colonization and infection.

## Main text

### Methods

#### Bacterial cultivation

In this study, fifty-three phyllosphere bacteria isolates were recovered from Guava (*Psidium guajava*), Starfruit (*Averrhoa carambola*), Madeira Vine (*Anredera cordifolia*) leaves from previous study [8, 9]. It were collected from Pondok Bambu and Karanganyar, Indonesia. For isolation of phillosphere bacteria, the leaves were put into the tubes containing 10 mL of phosphate buffer 10 mM pH 8. The tubes then put into sonicator for 5 min to release the bacteria from the leaves and homogenized into the buffer. Then, the bacterial suspensions were diluted using phosphate buffer with serial dilution started from 10^−1^ to 10^−4^. As much as 100 µL of the suspension were spread onto BHIA (Oxoid, Basingstoke, United Kingdom) and incubated at 28 °C for 48 h. All of the bacterial isolates from previous studies were refreshed onto King’s B 10% Agar (20 g Protease Peptone; 1.5 g K_2_HPO_4_; 1.5 g Mg_2_So_4._ 7H_2_O; 10 mL Glycerol; 10 g Agar Bacto; 1 L distilled water) and incubated at 28 °C for 48 h.

In this study we used several fish pathogenic bacteria including *S. agalactiae* ATCC27956, while *V*. *harveyi* and *A. hydrophila*. They were obtained from Health Aquatic Organism Laboratory, Department of Aquaculture, Faculty of Fisheries and Marine Sciences, Bogor Agricultural University. Both bacteria *V*. *harveyi* and *A. hydrophila* were isolated from infected fish and characterized with biochemical assays. For growing the *A. hydrophila,* it was streaked onto Luria Agar (5 g NaCl; 5 g Yeast extract; 10 g Tripton; 20 g Agar Bacto; 1 L distilled water) (LA) and incubated at 28 °C for 24 h. *V. harveyi* was streaked onto Luria Agar where concentration of NaCl is 2% and incubated at 28 °C for 24 h. *S. agalactiae* was streaked onto Luria Agar and incubated at 37 °C for 24 h.

#### Screening for anti-quorum sensing activity

All of the phyllosphere bacteria isolates were streaked onto King’s B 10% Agar and incubated at 28 °C for 48 h. While for *C. violaceum* as indicator strain were grown separately in 5 mL of Luria Broth and incubated at 28 °C overnight with an orbital shaker at 120 rpm. The monitor strain (100 µL) from a culture with OD_600_= 0.132 was added to semisolid agar (0.75% agar) of 7 mL volume for an overlay on top of the isolates. These plates were incubated at 28 °C for 24 h [10].

#### Production of crude extract

Phyllosphere bacteria isolates were inoculated in 100 mL of Luria Broth and incubated at 28 °C for 48 h using orbital shaker incubator. After that, the suspension of bacteria was centrifuged at 13,888×*g* for 15 min and then the supernatant was mixed with an equal volume of ethyl acetate (SmartLab) and it were kept in a rotary shaker 120 rpm overnight. The solvent layer was harvested and evaporated by a rotary evaporator, while the remaining solvent was evaporated using a vacuum oven overnight. After the extract were free from solvent, it was weighed and dissolved with 1% of DMSO (Smart Lab) with the final concentration reached at 20 mg/mL (w/v), then kept in the freezer − 20 °C [11].

#### Antibacterial activity assay

As much as 100 µL of pathogenic bacteria with OD_600_ = 0.132 were streaked continuously onto Brain Heart Infusion Agar (BHIA) (Oxoid, Basingstoke, United Kingdom) using sterile cotton buds. Then, the sterile cork borer was used to make a hole and filled by 100 µL of extract. In this study as positive control we used 10 mg/mL of streptomycin antibiotic (Merck), while 1% of DMSO was used as negative control. These plates were then incubated at 28 °C (*A. hydrophila* and *V. harveyi*) and 37 °C (*S. agalactiae*) for 24 h, this assay was performed in triplicate. Determination of Antibacterial activity were done through the clear zone performed as growth inhibition of fish pathogenic bacteria [12].

#### Detection of quorum quenching activity

*Chromobacterium violaceum* as indicator strain was added to BHIA and streaked continuously using sterile cotton buds. After that, similar with antibacterial activity assay, a hole were made using sterile cork borer and then filled with 100 µL of phyllosphere bacterial extract. Streptomycin (10 mg/mL) also used as positive control, for negative control we used 1% of DMSO. Incubation were done at 28 °C for 24 h. This assay was performed in triplicate. Determination of quorum sensing inhibition were done through formation of clear zone around the extract which indicated inhibition of violacein pigment production [10].

#### Quantification of anti-biofilm activity

In this assay we conducted two analysis for each extract which were inhibition or destruction of biofilm formed by pathogenic bacteria. For the inhibition assay, 100 µL of pathogen culture and 100 µL of the extract were transferred into 96 wells microplate. For positive control we used only pathogen. While for negative control we used BHIB (Oxoid, Basingstoke, United Kingdom) medium without pathogens. Microplates were then incubated at 28 °C and 37 °C overnight. For the destruction assay, 100 µL of pathogen culture were transferred into 96 wells microplate and incubated overnight. After incubation, we added 100 µL of the phyllosphere extracts into the microplate followed by incubated overnight. Planktonic cells and media were discarded after incubation, and adherent cells were rinsed with deionized water two times and then kept for 30 min for air-dried. Staining were done by using 200 µL of 0.4% crystal violet for 30 min to stain the biofilm adhere on microplates. After that the dye was discarded and rinsed five times with deionized water, and kept it air dried for 30 min. As much as 200 µL of 96% of ethanol was added into each well and resuspended. Then, 150 µL of 96% of ethanol from each well was transferred into a new microplate. The optical density was determined at 595 nm with microplate reader (TECAN M200 PRO) [13]. The percentage of inhibition is calculated using the following formula [13]:

$$ \% {\text{inhibition}}/{\text{destruction = }}\frac{{{\text{OD Control}} - {\text{OD Sample}}}}{{\text{OD Control}}} \times 100\% $$

### Results

#### Screening for anti-quorum sensing activity

**Fig. 1:**
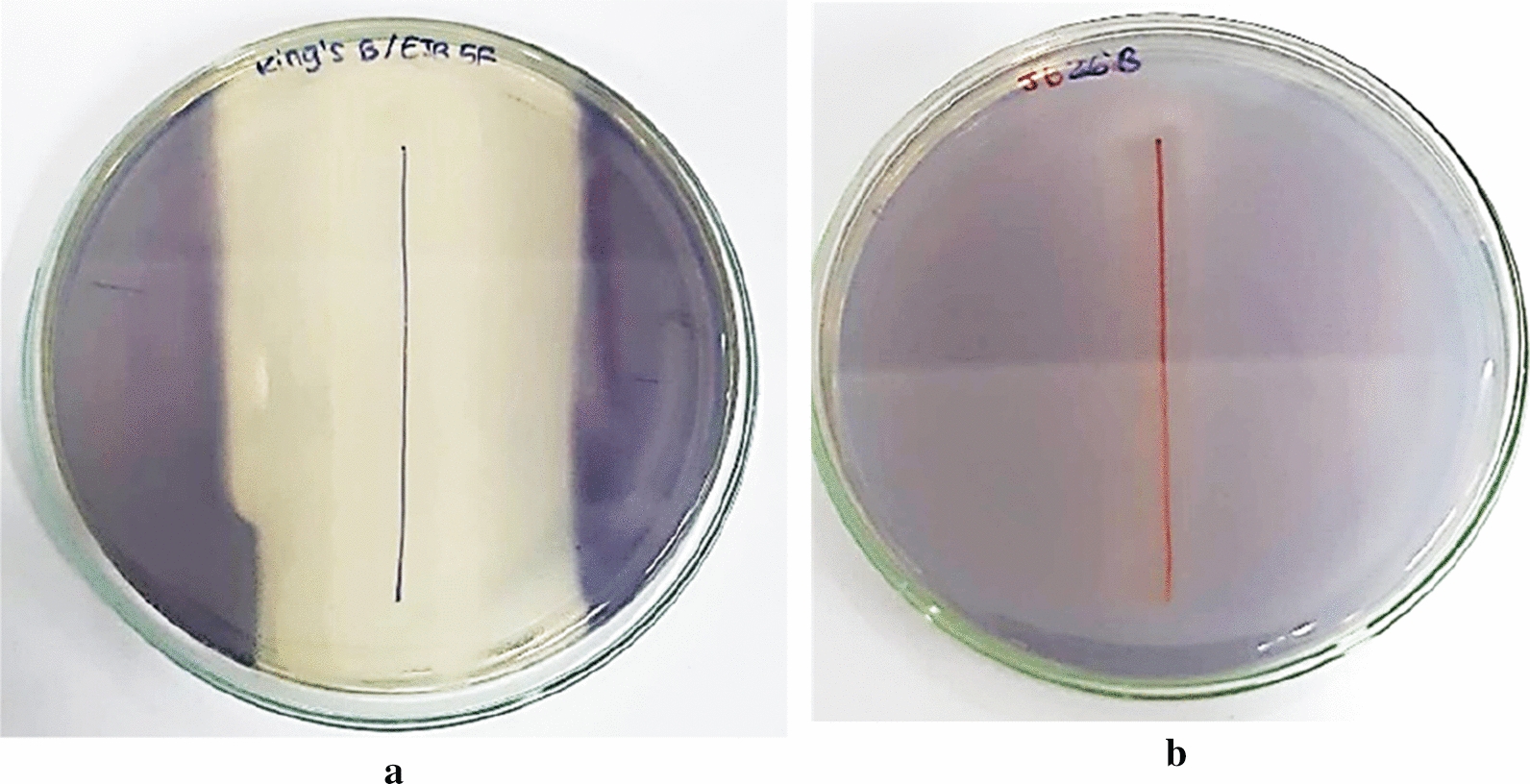
Quorum quenching activity and growth inhibition of phyllosphere bacteria isolates EJB 5F (**A**), the QQ activity is indicated by loss of the violet color of *C. violaceum*. In (**B**) QQ activity of isolate JB 26B

Screening of anti quorum quenching activity were conducted for all of 53 isolates. We recovered 34 out of the 53 phyllosphere bacteria isolates performed inhibition of violacein pigment production through determination of indicator strain. From these results we found that that phyllosphere bacteria isolates have quorum quenching activity against *Chromobacterium violaceum* (Fig. 1).

#### Antibacterial activity assay

**Fig. 2:**
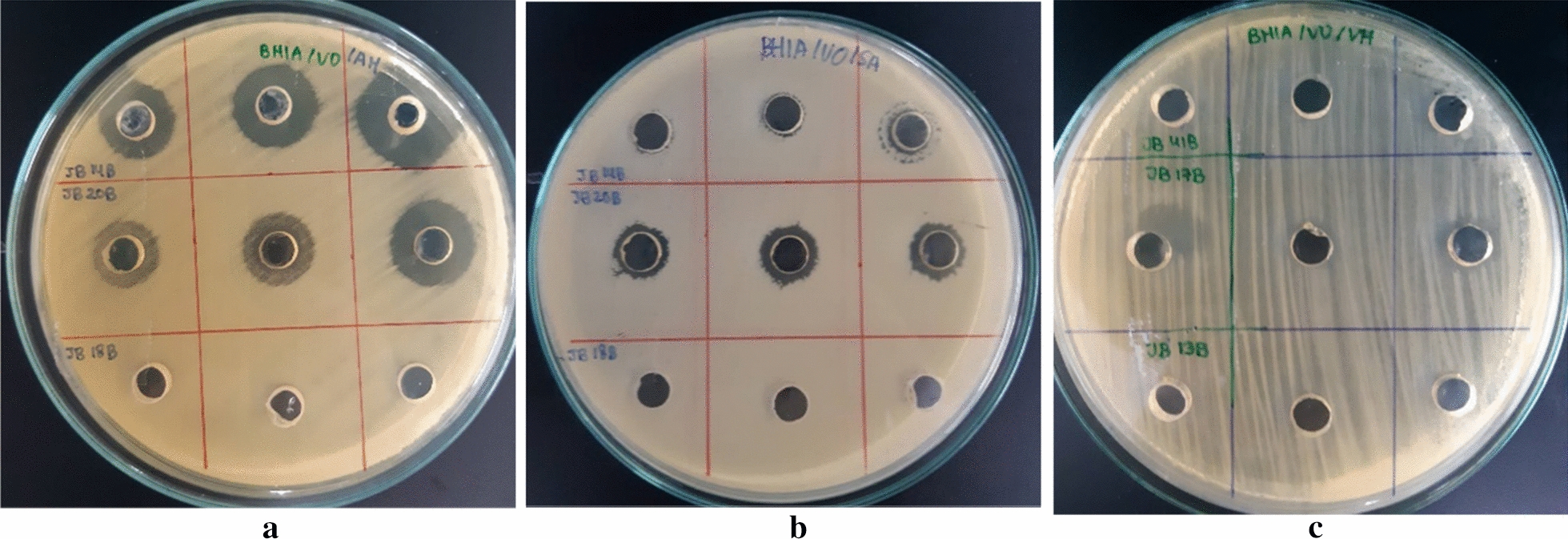
A clear zone of different phyllosphere bacteria, the activity was tested in the extract of each isolates against fish pathogens. **a** Antibacterial activity of isolates JB 14B and JB 20B against *A. hydrophila* (**b**) Antibacterial activity of isolate JB 20B against *S. agalactiae* (**c**) Antibacterial activity of isolate JB 17B against *V. harveyi*

Antibacterial Activity assay performed that isolates JB 14B and JB 20B showed antibacterial activity against *A. hydrophila*. Isolate JB 14B, JB 16B, and JB 17B have antibacterial activity against *V. harveyi*. Isolate JB 20B, JB24B, and EJB 5F have antibacterial activity against *S. agalactiae* (Fig. 2)

#### Detection of quorum quenching activity

From this assay, we found that 8 isolates from *P. guajava* form clear zone around the extract which indicate inhibition of pigment production. This result showed that the crude extracts have quorum quenching activity against *Chromobacterium violaceum*.

#### Quantification of anti-biofilm activity

The result of inhibition activity assay showed that crude extracts from isolate JB 17B showed the highest activity to inhibit biofilm formation of *A. hydrophila* with the result up to 86%. While isolate EJB 5F showed the highest inhibition activity in inhibiting biofilm formation of *V. harveyi* with the result up to 72%. Isolate JB 3B showed the highest activity to inhibit biofilm formation of *S. agalactiae* with the result up to 81% (Table 1).

**Table 1:** Biofilm inhibition and destruction activity against fish pathogen bacteria

Crude Extract	*A.hydrophila*		*V. harveyi*		*S. agalactiae*	
	% inhibition	% destruction	% inhibition	% destruction	% inhibition	% destruction
JB 3B	29	39	23	29	81	55
JB 16B	37	56	X	X	73	51
JB 17B	86	72	X	X	70	73
JB 20B	X	X	55	84	X	X
JB 26B	24	28	41	32	52	39
JB 8F	78	81	58	75	62	52
JB 12F	-	39	65	39	79	60
EJB 5F	42	29	72	41	X	X

NB: X = contains anti-bacterial activity,—= no activity

From destruction assay, there were several isolates that performed high activity to destruct biofilm of one specific fish pathogenic bacteria. Extract from isolate JB 8F showed the highest activity to disrupt biofilm of *A. hydrophila* (81% of destruction). Isolate JB 20B performed the highest activity in disrupting biofilm formed by *V. harveyi* with the percentage destruction of 84%. While isolate JB 17B also showed the highest activity to disrupt biofilm of *S. agalactiae* with the result up to 73% (Table 1).

### Discussion

Thirty-four isolates performed quorum quenching activity to inhibit the production of violacein pigment. Mechanism of quorum quenching were defined as inhibition of cell-to-cell communication through signal molecule interference, by using *C. violaceum*, these activity were determined in inhibition of violacein production [10].

Antibacterial activity assay showed that two out of fifty-three phyllosphere bacterial extracts inhibited the growth of *A. hydrophila*, while three out of fifty-three phyllosphere bacterial extracts performed antibacterial activity to *V. cholerae* and *S. agalactiae*. Those extracts with antibacterial activity were not continued for further assays, since it might cause false positive results in assessment of antibiofilm activity [12].

Several extracts showed quorum quenching activity due to inhibition of violacein pigment production, further studies were required to confirmed and determined the mechanism of bacterial communication inhibition, there are some possibilities might occurred which are inhibition of signal molecule production or interference of signal receptor [10].

The antibiofilm activity of phyllosphere bacterial extracts might come from their competencies to produce extracellular polymeric substance (EPS)-degrading enzyme. Both EPS production and biofilm formation are regulated by quorum sensing mechanism, which include production, release, as well as detection of signalling molecules. When population density are saturated it start induction of genes related with biofilm differentiation and maturation [14].

Inhibition of biofilm formation activity of phyllosphere bacteria might reflect quorum quenching activity of the phyllosphere bacteria extract to impede cell-to-cell communication of fish pathogenic bacteria used in this study. On the leaves surface there are several pressure which is need to be confronted by the microbe live in these habitats including nutrient limitation and environmental stress and physical condition. Hence, it is important for the bacteria in these habitat to develop adaptation and competitiveness to compete with other bacteria, one of them is capability to inhibit communication of other bacteria as well as production of extracellular polysaccharide and pigment to support adhesion on leaves surface also protection from other bacteria [15, 16].

There are several steps in the process of biofilm production in *A. hydrophila.* On the first step it were include production of lipopolysaccharides (LPS), and other surface polysaccharides (α-glucan), Mg^2+^ transporters, cytoskeletons as well as flagella and chemotaxis. This bacteria used.

AI-1 system to induce biofilm production and virulence-associated factors. The virulence factors regulated through this system were cover several virulence factors such as expression of hemolysin, metalloproteases, amylase, serine proteases, production of S-layer DNAase expression, including production of pigment.

There are two types of flagella (polar and lateral) is harbored by *A. hydrophila,* these two flagella are important for swimming and swarming and also play role in pathogenesis and biofilm formation of this bacteria [17, 18, 19]. According to Rasch et al. [20], the 3-oxo-C10-HSL produced by *Vibrio anguillarum* inhibits protease activities of *A. hydrophila*. Similarly, Ponnusamy et al. [21] showed that the synthetic 2(5H)-furanone derived from inhibitor AHL produced by *Delisea pulchra*, exhibited quorum quenching activity against C4-HSL and C6-HSL.

Biofilm formation of *V. harveyi* is under control of AHL-mediated QS mechanism. So far there are three molecules signals involved in quorum sensing mechanism of this bacteria, such as Harveyi autoinducer (HAI-1), Autoinducer 2 (AI-2), and Cholerae inducer 1 (CAI-1) [22]. There are positive correlation of biofilm formation with increasing in EPS production.

It was reported that the extract of *Synechococcus* sp interfere production of EPS. The GC–MS assays performed fatty acid and bioactive compound hexadecenoic acid [23], Similarly, based on Kannan et al. [24], biosurfactant from *Vibrio natriegens* which identified as glycolipid showed aptitude to reduce biofilm formation, bioluminescence, EPS, as well as quorum sensing of *V. harveyi*.

According to Rosini et al. [25], pili formed by *S. agalactiae* are important in biofilm formation of this bacteria and the gene encode GBS pilus machinery are clustered in three related genomic islands (Islands PI-1, -2a, and -2b. These gene encode protein which is contribute for arrangement of pilus while other two gene encode enzyme called sortase which is important for make covalent linkage of the pilus protein into the polymers.

Based on Rinaudo et al. [26] pilus type 2a are contribute in biofilm formation, in the absence or low expression of pilus 2a condition conduce decrease of biofilm production. On the other hand, sortase enzyme also hinder biofilm formation.

Destruction activity of extract from phyllosphere bacteria might come from the production of matrix-degrading enzymes. EPS-degrading enzymes promote degradation of extracellular matrix as one of the major component in biofilm. Compare with other enzymes, protease known potential to destruct biofilm, since the major component of extracellular polymeric substance are proteins [27].

Based on antibiofilm activity results, both inhibition and destruction showed that not all of the extracts have both activities. *V. harveyi* and *S. agalactiae* biofilm could be inhibited by all extracts of isolates, meanwhile biofilm of *A. hydrophila* could not be inhibited by isolate JB 12F. This might be caused by the lack of enzyme production to inhibit the biofilm component of *A. hydrophila*. Furthermore, the variety of the result might come by several factors, including different component of biofilm formed by pathogenic bacteria, different compound in the extracts itself both for biofilm disruption and inhibition, environmental factors (pH and temperature) which affect the biofilm formation [28].

### Conclusion

In this study we found eight extracts from phyllosphere bacteria with quorum quenching activity. All of these extracts are also capable to inhibit and disrupt biofilm formed by fish pathogenic bacteria. It is potential to be applied for prevention and treatment of infectious diseases caused by pathogenic bacteria used in this study.

Further research is important to be conducted to explore application of phyllosphere bacteria extract in the aquaculture ecosystem. However, other assays are needed to be carried out such as validation of quorum quenching molecules, molecular identification of quorum quenching, biofilm destruction molecules, and identification of potential bacteria to support this research. In addition, a combination assay of different extracts also required to be explored which might vary in the effectiveness.

## Limitations

The pathogen bacteria that we selected represents pathogens commonly found in fish. Activity of the extract with other fish pathogenic bacteria might be different and need to be explored for further study. The main component of the extract has not been identified although many studies describe the major component is protein. Quantitative assay for quorum quenching activity need to be done for further study as well as Scanning Electron Microscopy observation to get image on how the biofilm destruction process.

## Data Availability

The datasets used and/or analysed during the current study are available from the corresponding author on reasonable request.

## Abbreviations

EPS: Extracellular polymeric substances
AHL: Acyl homoserine lactone
